# The Design and Evaluation of an Online Continuing Medical Education App for Medical Professionals in China: Quantitative Study

**DOI:** 10.2196/76299

**Published:** 2026-02-23

**Authors:** Xu Zhang, Xianying He, Yuntian Chu, Dongqing Liu, Minzhao Lyu, Weiyi Wang, Haotian Chen, Meihao Ji, Fangfang Cui, Jie Zhao

**Affiliations:** 1 National Engineering Laboratory for Internet Medical Systems and Applications, The First Affiliated Hospital of Zhengzhou University, 1st Jianshe Road, Zhengzhou, 45002, China, 8637167966286; 2 School of Electrical Engineering and Telecommunications, University of New South Wales, Sydney, Australia; 3 Institute of Intelligent Medicine, Henan Academy of Innovations in Medical Science, Zhengzhou, China

**Keywords:** online continuing medical education, mobile medical education, trend, satisfaction, ability enhancement

## Abstract

**Background:**

As an emerging delivery mode of education, online continuing medical education (CME) increases the accessibility of high-quality medical training for professionals and students in China. Guoyuan (meaning “nationwide” in Chinese) is an online CME platform delivered via a mobile app and operated by the National Telemedicine Center of China since 2018, serving as an illustrative case of mobile online CME implementation.

**Objective:**

We identified trends in the adoption and usage of the Guoyuan mobile online CME platform from 2018 to 2023 and provided evidence for the application and optimization of online CME.

**Methods:**

We analyzed yearly usage data of the Guoyuan mobile app (The First Affiliated Hospital of Zhengzhou University) in 2018-2023 and collected surveys on the satisfaction and recognition of competency enhancement in online CME in each connected hospital in 2023. Using the IBM SPSS, the nonparametric Kruskal-Wallis *H* test was used to compare attendance across different disciplines, followed by post hoc pairwise comparisons for course types with significant differences and ordinal logistic regression analysis to examine factors influencing satisfaction with the online CME system and perceived competency enhancement among invited doctors.

**Results:**

From 2018 to 2023, Guoyuan had 94,537 registered trainees, 1672 published course videos, and 1,878,437 attendances. Attendance was higher for courses in ophthalmology, otolaryngology, and pathology than in other disciplines (median attendance 610, IQR 105-2055 vs 283, IQR 106-690 participants). Based on a sample size of 245 participants, ordinal regression analysis showed that discipline category, professional title, and working years significantly influenced satisfaction. General practitioners showed lower overall satisfaction than internal medicine doctors (odds ratio [OR] 0.323, 95% CI 0.110-0.948; OR 0.251, 95% CI 0.087-0.729; and OR 0.196, 95% CI 0.066-0.585; *P*=.04; *P*=.01*; P*=.003). Junior titles reported higher audio-visual clarity (OR 3.151, 95% CI 1.178-8.427; *P=*.02) and process satisfaction (OR 4.939, 95% CI 1.674-14.576; *P*=.004). More experienced doctors had higher system usability (OR 1.102, 95% CI 1.012-1.200; *P*=.03) and process satisfaction (OR 1.141, 95% CI 1.044-1.247; *P*=.003). Recognition of online CME’s benefits was influenced by multiple factors. Greater clinical experience positively predicted recognition of clinical use (OR 1.106, 95% CI 1.004-1.218; *P*=.04), while an inverse association was observed with age (OR 0.894, 95% CI 0.802-0.996; *P*=.04). For research-related benefits, positive predictors included discipline category in obstetrics and gynecology compared to internal medicine (OR 6.217, 95% CI 1.236-31.258; *P*=.03) and junior professional title (OR 3.791, 95% CI 1.231-11.673; *P*=.02), whereas intensive care unit was a negative predictor compared to internal medicine (OR 0.111, 95% CI 0.014-0.893; *P*=.04).

**Conclusions:**

Online mobile CME platforms have gained widespread adoption among medical professionals in China, particularly after the COVID-19 outbreak. However, substantial disciplinary disparities in course availability and user experience persist, indicating the need for further optimization of course design and software interaction.

## Introduction

The unbalanced distribution of medical resources is a critical issue that significantly impacts the overall well-being of citizens, particularly in low-income countries [1]. This problem is even more acute in China, which has a population of more than 1.4 billion, with approximately 491 million citizens residing in rural areas that are currently experiencing shortages of high-quality medical resources [2,3]. Although the Chinese government is actively seeking effective measures to increase the coverage of medical resources [4], the issue of unbalanced distribution remains unsolved. In 2017, the number of physicians per 1000 population in eastern, middle, and southern China was 4.3, 3.8, and 3.5, respectively [5,6]. Prior research [7] has used the health resource agglomeration degree to describe the qualification of medical professionals. In 2018, the health resource agglomeration degree value for urban regions in China was 1.60 (sufficient), while the value for rural regions in China was 0.68 (severely insufficient). Furthermore, the annual growth rate for the number of medical professionals in rural regions is much lower than that in urban regions, making the problem increasingly severe over the years [8-10].

Continuing medical education (CME) is a critical component of medical education, aimed at enabling health care professionals to continuously acquire new knowledge and innovative technologies relevant to their specialties. It serves as a continuation, supplement, and enhancement of professional education, ultimately enhancing the technical competencies and service quality of medical practitioners. Without a program of active learning, no physician will be able to remain competent for more than a few years after graduation [11]. Common CME formats include lectures, conferences, printed materials, internet-based educational activities, and open discussions [12]. Online CME is one of the technologies aiming to provide accessible medical training and education for medical professionals at less-privileged hospitals in rural and remote regions without requiring them to physically attend classes held by leading institutes, which can be impractical due to financial and physical constraints [13]. As reported by prior research, online CME has gained popularity for medical education on an international scale [14-21]. During the COVID-19 pandemic, it has become a major form of medical education across many disciplines, such as anatomy and physiological classes in universities in Australia and New Zealand [17]. Interestingly, survey studies conducted in the United States and Australia [18,19] suggest that students and trainees in online anatomy and physiotherapy courses have reported better learning efficiency via online CME than in-person classes.

To the best of our knowledge, existing research primarily focuses on online learning among university students, while CME for health care professionals is mainly concentrated on discipline-specific training. In contrast, there is relatively little research on multidisciplinary and comprehensive CME for medical professionals. The increasing burden of chronic diseases in China has led to a growing demand for high-quality primary health care services. Online CME has a positive impact on physician performance, especially for primary care doctors. However, many primary health care professionals in China perceive a lack of online CME opportunities and have low participation rates, despite having high expectations [22]. A mobile online education model that enhances the accessibility of digital learning, facilitates large-scale implementation, and strengthens the capacity of primary health care systems urgently warrants broader promotion and application.

In this study, by introducing the Guoyuan platform (meaning “nationwide” in Chinese) as an illustrative example of a mobile online CME app serving hospitals in Henan, China’s most populous province, we propose a mobile online CME paradigm. Through this case, we demonstrate a model where educators disseminate knowledge across online channels, enabling learners to access educational content on demand by using fragmented time. By analyzing data from the Guoyuan platform spanning 2018 to 2023, together with questionnaire responses regarding online CME, this study seeks to investigate the platform’s curriculum characteristics and identify the key factors influencing doctors’ satisfaction and perceived competence improvement. Through these analyses, this study intends to provide a viable online CME model for developing regions and offer empirical evidence for the optimization of future online education programs, with the ultimate goal of reducing urban–rural disparities in health care resources and enhancing the professional competence of primary health care personnel.

## Methods

### The Guoyuan Mobile Online CME Platform

The Guoyuan mobile online CME platform is developed by the National Telemedicine Center of China and currently serves medical professionals in both premium tier-3 and less-capable tier-2 and tier‐1 hospitals primarily within Henan province (China is currently categorizing medical institutes into 3 tiers. In general, tier-3 hospitals, being the highest rank, are large-scale comprehensive or specialized medical centers at the top of the health care system; tier-2 hospitals function as regional medical centers focusing on common and frequently occurring diseases, while tier-1 hospitals are community health centers providing basic care, prevention, and rehabilitation services. The service coverage will be expanded to the entire nation in the next deployment phase of the platform. It delivers medical training and courses instructed by professors and highly qualified medical professionals in the tier-3 hospitals. Registered trainees in connected hospitals could access the online courses via an Android and iOS mobile app specifically designed for the platform. To motivate course instructors for high-quality content, enrollment fees are charged on a per-course basis, paid by the connected hospitals or individual trainees.

**Figure 1.**
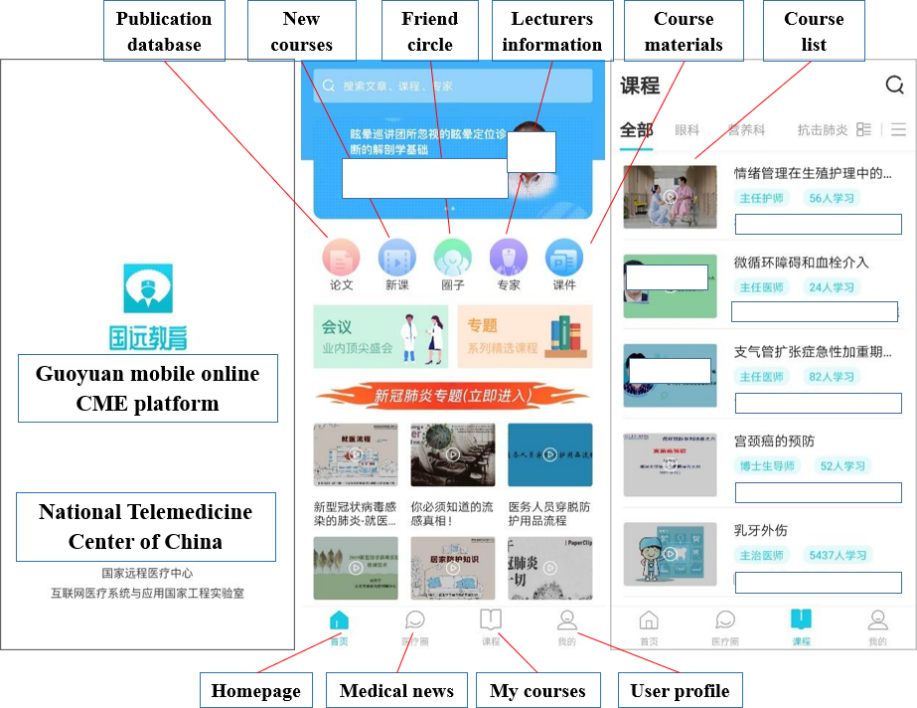
Three example user pages of the Guoyuan mobile online continuing medical education app. CME: continuing medical education.

Within the client mobile app, after login and initialization, trainees could access a range of services, including their courses in progress, newly released courses under different disciplines, lecturer information, course materials, and publication database, as visually shown in Figure 1. For each available course, in addition to scheduled live lectures that allow trainees to interact with course instructors in real time, lecture recordings and discussion forums are also available to trainees for after-class reviews and discussions. In addition to the core functions in online learning, a registered user could also have their friend circles socialize with colleagues in other medical institutes. Detailed descriptions of each available module on the mobile app are given in Multimedia Appendix 1.

### Data Collection

The dataset used in this study comprises the usage data of the regional online CME platform delivered via the Guoyuan mobile app over 6 years from 2018 to 2023. The scope of the dataset contains course metadata (eg, names, disciplines, instructors, sessions, enrollments, and attendance), connected hospitals, and registered medical professionals. In addition, to understand the satisfaction and recognition of competency enhancement in online CME, we created an online questionnaire. According to the sample size calculation formula as follows:


(1) $$n_{0}=\frac{Z_{\alpha /2}^{2}\;p(1-p)}{\delta^{2}}\;n=\frac{n_{0}}{1+\frac{n_{0}-1}{N}}$$

Where $n_{0}$ represents the initial sample size calculated for an infinite population, $Z_{\alpha /2}^{2}$ represents 95% CI, p represents an estimated population proportion of 0.5, and $\delta^{2}$ represents an acceptable margin of error of 0.1, N represents the total number of registered users on the Guoyuan app, and accounting for a 10% invalid response rate, the final calculated sample size was 108 participants. Based on the proportion of registered users by hospital level, the sample included 48 participants from tier-3 hospitals and 60 from tier 2 and tier 1 hospitals. The survey notice was distributed via the Guoyuan app to hospital administrators across the province, who were responsible for organizing local participation. The questionnaire was completed using WJX (an online survey software developed by a Chinese company). In total, we obtained 245 valid questionnaires, including 98 from tier 3 hospitals and 147 from tier 2 hospitals. The questionnaire encompassed 3 core sections: doctors’ demographic information (eg, age, sex, professional title, education, and working experience), institutional profiles of their affiliated hospitals (eg, province, levels of hospital and which discipline the provider was from), and their satisfaction levels with online CME, alongside perceived competency enhancement through the online CME.

### Statistical Analysis

The dataset we have collected consists of system logs that require data cleaning and preprocessing, which are performed using utilities in the Excel software (Microsoft Corp). Our analysis on the cleaned intermediary dataset leveraged SPSS software (version 25.0; IBM Corp). Absolute values, fractional ratios, and statistical metrics such as median, mean values, and total values were used to describe the adoption of courses under different medical disciplines, their enrollment and attendance, and annual changes. In the line and bar charts, “yearly published courses,” “average attendance,” and “total attendance,” indicate the number of newly released courses and their corresponding attendance for each year from 2018 to 2023. “Number of published courses,” “median attendance per course,” and “total attendance” shown in the table described the cumulative data from 2018 to 2023. The nonparametric Kruskal-Wallis *H* test was used to compare attendance across different disciplines, followed by post hoc pairwise comparisons for course types with significant differences. An ordinal logistic regression analysis was applied to examine the impact of hospital level, region, department classification, gender, and professional title [23] on satisfaction with the convenience of the online CME system, audio-video quality, and process. Furthermore, using data from inviting doctors, an ordinal logistic regression analysis was used to analyze the factors influencing the effectiveness of online CME in enhancing the skills of inviting doctors. The significance level was set at α=.05.

### Ethical Considerations

This study was approved by the First Affiliated Hospital of Zhengzhou University (Ethical Code: 2023-KY-0488-002). The data from the Guoyuan app was limited to course-related statistics, such as the number of registrations and the number of course views, and the data collected from the questionnaire were limited to demographic information, such as gender and age. No sensitive or personally identifiable biological information was collected in any of the data. The doctors were aware that the consent information was uploaded to the National Telemedicine Center of China and agreed to the data analysis and utilization. Informed consent was obtained from all individual participants involved in the study. For secondary analyses of research data, we confirm that the original informed consent or institutional review board approval included provisions allowing for secondary analysis without additional consent from the participants. Participants in the study were not offered any form of compensation for their participation. No images of individual participants or users are included in the manuscript or supplementary material that would allow for identification.

## Results

### User Popularity

Guoyuan mobile online CME platform was launched in 2018, with cumulative registrations reaching 94,537 as of 2023. The annual registered users in 2018, 2019, 2020, and 2021 were 4902, 7879, 23,940, and 47,148 persons, respectively, demonstrating a marked growth trend with an average annual growth rate of 112.7%. The fastest growth occurred in 2020, with a year-on-year increase of 203.9% (23,940 in 2020 compared with 7879 in 2019), while the highest number of registrations was in 2021, reaching 47,148, with a year-on-year growth of 96.9% (47,148 in 2021 compared with 23,940 in 2020). From 2021 to 2023, the number of registrations showed a downward trend.

### Available Courses and Attendance

**Figure 2.**
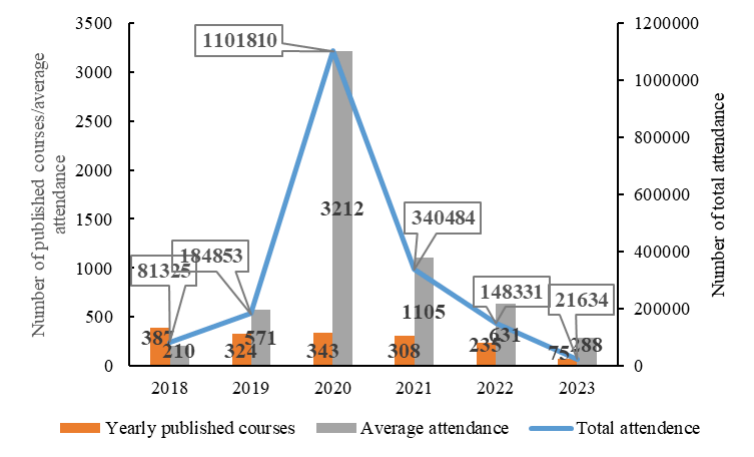
Number of available courses and their attendance.

During the period of 6 years from 2018 to 2023, a total of 1672 courses were published on the online CME platform. The total attendance for all courses during this period is 18,78,437 persons. The yearly numbers of published courses are depicted as the orange bar in Figure 2. In addition, the figure also shows the yearly course attendance represented by both aggregated count and averaged count per course, as annotated by the blue lines and gray bars, respectively. Notably, due to the COVID-19 outbreak, the total attendance in 2020 reached an abruptly high value of 1,101,810 persons, a 496.1% increase compared to 184,853 persons in 2019. From 2020 to 2023, the total attendance showed a downward trend, with the most significant decrease in 2023, which decreased by 85.4% (21,634 in 2023 compared with 148,331 in 2022). In general, the number of available courses remained roughly similar across years except for a significant decline in 2023. Both total attendance and average attendance peaked in 2020 before declining annually.

We analyzed the availability of courses and their attendance under different medical disciplines (Table 1). Internal medicine was found to be the most dominant discipline in terms of total attendance, with cumulative viewership reaching 915,754. This is not surprising, as the discipline had 728 out of 1675 courses published, accounting for 43.5% of all courses on the online CME platform.

**Table 1.** Availability of courses and their attendance under different medical disciplines (*H*=93.182; *P*<.001).

Disciplines	Number of published courses	Attendance per course (Q₁/Q₃), median (IQR)^a^	Total attendance
Pathology	105	283 (106-690)	98,247
Internal medicine	728	160 (36-914)^b^^,c^	915,754
Surgery	115	174 (48-712)^b^^,^^c^	102,911
Pediatrics	250	98 (26-804)^b^^,^^c^^,^^d^	121,186
Obstetrics and gynecology	121	94 (35-448)^b^^,^^c^^,^^d^	80,986
Ophthalmology and otolaryngology	103	610 (105-2055)	170,410
Traditional Chinese medicine	16	253 (180-940)	18,881
Pharmacy	71	70 (33-249)^b^^,^^c^^,^^d^	30,946
Nursing	88	104 (39-639)^b^^,^^c^	63,236
Others	78	407 (302-668)	282,964

a All comparisons were adjusted using the Bonferroni correction.

b Statistically significant difference compared to ophthalmology and otolaryngology.

c Statistically significant difference compared to others.

d Statistically significant difference compared to Pathology.

In terms of course availability, the second and the third popular disciplines were pediatrics and obstetrics and gynecology with their number of available courses as 250 out of 1675 (14.9%) and 121 out of 1675 (7.2%), respectively; whereas the second and the third popular disciplines ranked by their total attendance were ophthalmology and otolaryngology (170,410 views) and pediatrics (121,186 views). When comparing the median viewership across all disciplines, statistically significant differences were observed among disciplines (*H*=93.182, *df*=9; *P*<.001). Post hoc pairwise comparisons of course viewership revealed that the median viewership for pathology was 283, which was higher than that of pediatrics, obstetrics and gynecology, and pharmacy (*P*=.009; *P*=.006; *P*=.003). The median viewership for ophthalmology and otolaryngology and other disciplines (eg, infection management, telemedicine, etc) was 610 and 407, respectively, which was higher than that of internal medicine, surgery, pediatrics, obstetrics and gynecology, pharmacy, and nursing (*P*<.001).

### Online CME Satisfaction Evaluation

According to the evaluation from the survey, 233 out of 245 (95.1%) doctors, 224 out of 245 (91.4%) doctors, and 233 out of 245 (95.1%) doctors expressed satisfaction with system usability, audio and video clarity, service process, respectively. Using an ordinal regression model, we analyzed the impact of hospital level, region, professional title, age, discipline category, working years, telemedicine working years, and other variables on online CME satisfaction. The results (Table 2) showed that the discipline category was a factor influencing satisfaction across 3 dimensions. General practitioners had lower satisfaction in all 3 aspects of online CME compared to internal medicine doctors, with an odds ratio (OR) of 0.323 (95% CI 0.110-0.948), 0.251 (95% CI 0.087-0.729), and 0.196 (95% CI 0.066-0.585), respectively (*P*=.003).

**Table 2.** Ordinal logistic regression analysis results for satisfaction with online continuing medical education.

Characteristics	Satisfaction degree toward system usability		Satisfaction degree toward audio and video clarity		Satisfaction degree toward service process	
OR (95% CI)^a^	*P* value	OR (95% CI)^a^	*P* value	OR (95% CI)^a^	*P* value
Hospital level (ref: tier-3)						
Tier-2 and tier‐1	1.897 (0.976-3.688)	.06	1.330 (0.691-2.560)	.39	1.356 (0.684-2.692)	.38
Region (ref: Western)						
Eastern	0.474 (0.188-1.195)	.11	1.041 (0.424-2.557)	.93	1.596 (0.611-4.164)	.34
Central	0.888 (0.434-1.816)	.75	1.107 (0.558-2.197)	.77	1.199 (0.584-2.463)	.62
Discipline (ref: Internal medicine)						
Others	0.511 (0.156-1.671)	.28	0.759 (0.234-2.462)	.65	1.033 (0.279-3.830)	.96
Surgery	0.833 (0.383-1.810)	.64	1.175 (0.554-2.492)	.67	1.341 (0.605-2.974)	.47
Pediatrics	0.903 (0.258-3.162)	.87	1.648 (0.453-6.001)	.50	1.652 (0.452-6.039)	.45
Obstetrics and gynecology	0.661 (0.218-2.005)	.47	0.632 (0.216-1.848)	.40	1.239 (0.372-4.126)	.73
Pathology	1.739 (0.433-6.979)	.44	2.245 (0.547-9.221)	.26	1.969 (0.489-7.936)	.34
General medicine	0.323 (0.110-0.948)	.04^b^	0.251 (0.087-0.729)	.01^b^	0.196 (0.066-0.585)	.003^b^
Traditional Chinese medicine	0.541 (0.096-3.056)	.49	0.506 (0.119-2.153)	.36	0.492 (0.095-2.545)	.40
Intensive care unit	0.151 (0.024-0.949)	.04^b^	1.645 (0.232-11.646)	.62	1.306 (0.176-9.663)	.80
Sex (ref: Female)						
Male	0.795 (0.440-1.435)	.48	0.671 (0.379-1.187)	.17	0.782 (0.429-1.425)	.42
Title (ref: Senior)						
Junior	2.619 (0.937-7.322)	.07	3.151 (1.178-8.427)	.02^b^	4.939 (1.674-14.576)	.004^b^
Intermediate	1.250 (0.574,2.724)	.57	1.940 (0.899-4.187)	.09	2.247 (0.994-5.080)	.05
Education (ref: PhD)						
Associate or below	0.586 (0.126-2.727)	.50	1.329 (0.308-5.730)	.70	1.583 (0.331-7.564)	.57
Bachelor	0.666 (0.188-2.359)	.53	1.098 (0.333-3.617)	.88	1.330 (0.371-4.770)	.66
Master	1.109 (0.311-3.960)	.87	1.489 (0.451-4.921)	.51	1.877 (0.518-6.800)	.34
Age	0.927 (0.848-1.014)	.10	0.931 (0.855-1.013)	.10	0.901 (0.823-0.987)	.03^b^
Working years	1.102 (1.012-1.200)	.03^b^	1.085 (0.999-1.177)	.05	1.141 (1.044-1.247)	.003^b^
Telemedicine working years	1.001 (0.937-1.068)	.10	1.013 (0.949-1.081)	.70	0.998 (0.931-1.070)	.97

a OR: odds ratio.

b *P* values <.05 are statistically significant.

In terms of system usability, working years and discipline category were factors affecting satisfaction. Doctors with more years of experience tended to have higher satisfaction with system usability, with OR 1.102 (95% CI 1.012-1.200; *P*=.03). Intensive care unit doctors had lower satisfaction with system operations compared to internal medicine doctors (OR 0.151, 95% CI 0.024-0.949; *P*=.04). Regarding audio-video clarity, professional title was a factor influencing satisfaction. Compared to senior titles, junior titles had higher satisfaction with the clarity of audio and video (OR 3.151, 95% CI 1.178-8.427; *P*=.02). For the service process, professional title, age, and working years were factors affecting satisfaction. Junior-level doctors reported higher satisfaction with the process than senior-level doctors, with OR 4.939 (95% CI 1.674-14.574; *P*=.004). Older doctors tended to be less satisfied with the process, with OR 0.901 (95% CI 0.823-0.987; *P*=.03). Those with more years of experience reported higher satisfaction, with OR 1.141 (95% CI 1.044-1.247; *P*<.003).

### Recognition of the Ability Enhancement Through Online CME

We collected evaluations from invited doctors on the recognition for improved diagnostic and treatment capabilities and recognition for enhanced research capabilities. The results (Table 3) showed that 236 out of 245 doctors (96.3%) recognized that online CME can enhance their clinical diagnosis and treatment abilities, while 213 out of 245 doctors (86.9%) believed it can improve their research capabilities. Using an ordinal regression model, we analyzed the impact of hospital level, professional title, region, discipline category, age, education level, and other variables on recognition of the ability enhancement through online CME. Regarding the recognition of online CME in enhancing clinical diagnosis and treatment capabilities, age and years of experience were influencing factors. Clinical experience, measured by working years, was a significant positive factor in recognizing the clinical benefits of online CME (OR 1.106, 95% CI 1.004-1.218; *P*=.04). An independent association with age was also observed (OR 0.894, 95% CI 0.802-0.996; *P*=.04). For the recognition of online CME in improving research capabilities, discipline category and professional title were key factors. Compared to internal medicine, obstetrics and gynecology doctors were more likely to recognize the research benefits of online CME, while intensive care unit doctors were less likely to do so, with an OR of 6.217 (95% CI 1.236-31.258; *P*=.03) and 0.111 (95% CI 0.014-0.893; *P*=.04) respectively. Additionally, junior-level doctors were more likely than senior-level doctors to believe that online CME can enhance research capabilities (OR 3.791, 95% CI 1.231-11.673; *P*=.02).

**Table 3.** Ordinal logistic regression analysis results for recognition of enhancing capabilities.

Characteristics	Level of recognition for improving diagnostic and treatment capabilities		Level of recognition for enhanced research capabilities	
	OR (95% CI)^a^	*P* value	OR (95% CI)^a^	*P* value
Hospital level (ref: tier-3)				
Tier-2 and tier‐1	1.531 (0.721-3.250)	.27	1.539 (0.750-3.155)	.24
Region (ref: Western)				
Eastern	1.656 (0.589-4.650)	.34	0.953 (0.350-2.591)	.93
Central	1.202 (0.557-2.594)	.64	1.226 (0.565-2.663)	.61
Discipline (ref: Internal medicine)				
Others	0.759 (0.155-3.730)	.73	1.501 (0.321-7.007)	.61
Surgery	1.952 (0.828-4.601)	.13	1.493 (0.671-3.319)	.33
Pediatrics	1.663 (0.363-7.626)	.51	1.364 (0.337-5.519)	.66
Obstetrics and gynecology	1.680 (0.482-5.857)	.42	6.217 (1.236-31.258)	.03^b^
Pathology	2.848 (0.513-15.814)	.23	2.649 (0.601-11.671)	.20
General medicine	0.335 (0.101-1.109)	.07	0.496 (0.166-1.489)	.21
Traditional Chinese medicine	0.368 (0.040-3.344)	.38	0.322 (0.051-2.026)	.23
Intensive care unit	0.614 (0.086-4.362)	.63	0.111 (0.014-0.893)	.04^b^
Sex (ref: Female)				
Male	0.803 (0.413-1.561)	.52	0.792 (0.420-1.493)	.47
Title (ref: Senior)				
Junior	1.451 (0.458-4.597)	.53	3.791 (1.231-11.673)	.02^b^
Intermediate	0.916 (0.389-2.157)	.84	1.655 (0.719-3.806)	.24
Education (ref: PhD)				
Associate or below	3.545 (0.588-21.354)	.17	1.120 (0.210-5.959)	.90
Bachelor	1.668 (0.387-7.185)	.49	1.063 (0.277-4.084)	.93
Master	1.991 (0.451-8.781)	.36	0.822 (0.207-3.269)	.78
Age	0.894 (0.802-0.996)	.04^b^	0.940 (0.851-1.038)	.22
Working years	1.106 (1.004-1.218)	.04^b^	1.091 (0.997-1.194)	.06
Telemedicine working years	0.956 (0.891-1.026)	.21	0.966 (0.897-1.039)	.35

a OR: odds ratio.

b *P* values <.05 are statistically significant.

## Discussion

### Main Findings

As the emerging paradigm in medical education, online CME has become increasingly popular in recent years, as evidenced by prior studies on its adoption by students in medical schools [24,25]. Our study demonstrates that online CME is also being widely adopted by medical professionals in hospitals, as seen in the Guoyuan mobile online CME platform by the National Telemedicine Center of China. This platform offers a wide range of functionalities, including live streaming, offline lecture recording, literature archives, and discussion forums, as well as a comprehensive selection of courses taught by professors from medical schools and tier-3 hospitals across various disciplines, which has been widely accepted by hospital staff in Henan Province, China. Since 2018, the number of registered users and the average course attendance have shown a rapid increasing trend, particularly after the outbreak of the COVID-19 pandemic, followed by a gradual decline returning to normal levels, which is consistent with the conclusions drawn by previous studies [26-28]. The attendance for courses in ophthalmology, otolaryngology, and pathology was higher than that in other disciplines, with a median attendance of 610 and 283 participants, respectively. Discipline category, professional title, and working years were the main factors influencing satisfaction and recognition of competency enhancement in online CME.

### Service Coverage and Popularity Among Medical Disciplines

The regional online CME platform offers courses across a wide range of medical disciplines. Internal medicine was the most popular in terms of course availability, total attendance, and average participation, likely reflecting its numerous subdisciplines and growing clinical priorities such as respiratory medicine and oncology, with courses on lung cancer, chronic obstructive pulmonary disease, gastric cancer, and liver cancer [29-31]. Ophthalmology and otolaryngology courses recorded the highest median views. These fields involve key facial regions, including the eyes, ears, and maxillofacial areas, and are among the fastest-growing specialties in digital technology integration. Rapid adoption of innovations such as implant navigation, 3D-printed jawbone prostheses, and artificial intelligence–assisted orthodontic design has shortened innovation cycles compared with general surgery [32,33]. The continuous emergence of new technologies requires practitioners in these fields to update their cross-disciplinary knowledge frequently, resulting in high attendance rates. However, as reported in a previous study [34], tier-2 and tier-1 hospitals often lack competence in surgery, pathology, medical imaging, and pharmacy. Our findings further indicate that these disciplines are insufficiently represented in the current online CME offerings, highlighting the need to expand course coverage and strengthen targeted training to enhance professional capacity in less-resourced hospitals.

### Influencing Factors and Optimization Strategies for Doctors' Satisfaction

Satisfaction with online CME varied across specialties and professional levels. General practitioners reported lower satisfaction than internal medicine doctors, likely due to limited hardware, slower networks, and outdated equipment in primary health care institutions compared to tertiary hospitals equipped with 5G systems [35]. Moreover, online CME content often emphasizes specialized knowledge, which mismatches the broader training needs of general practitioners, leading to cognitive overload and unmet expectations. Professional title also influenced satisfaction: junior doctors expressed higher satisfaction with audio-video quality and service process than senior doctors, as online CME aligns with their exam-oriented learning needs and digital-native familiarity with online systems [36]. Regarding system convenience and process satisfaction, doctors with more years of experience exhibited higher satisfaction. Senior doctors have witnessed the evolution of medical information systems over the years, gradually strengthening their technological adaptability and accumulating extensive experience in handling system errors, leading to higher tolerance for workflow disruptions. Overall, online CME satisfaction was relatively high, but general practitioners reported lower satisfaction levels. It is recommended to gather extensive feedback from general practitioners, increase relevant course offerings, and improve both the hardware infrastructure of primary health care institutions and software interaction experiences to further enhance the quality of online CME services.

### Influencing Factors and Optimization Strategies for Doctors' Recognition of Competency Enhancement in Online CME

Analysis of doctors’ recognition of online CME revealed that both age and years of experience significantly influenced perceptions of its value in improving clinical skills. Doctors with more years of experience tended to recognize its practical benefits, while age showed a modest negative effect. This may reflect a cognitive imbalance between confidence in personal experience and reliance on digital tools [37]. Their extensive clinical experience enhances their ability to judge the practicality of online CME content (a positive effect of experience), while their reluctance toward new technological platforms (a negative effect of age) creates a value perception conflict. In terms of research capability enhancement, junior doctors exhibited higher acceptance of online CME. Being in the early stages of research skill development, they often lack proficiency in literature retrieval and experimental design. Online CME effectively bridges these gaps, enhancing their confidence and competence. Overall, doctors demonstrated strong recognition of online CME’s contribution to professional growth, though some age-related resistance remains. Future course design should adopt an “age-friendly” and “experience-adaptive” approach, integrating senior experts’ practical knowledge with digital learning. Meanwhile, strengthening research-oriented modules aligned with career progression can further support junior doctors’ academic development.

### Trend Analysis and the Future of Online Mobile CME

During the 6-year period from 2018 to 2023, total attendance for online courses on the Guoyuan mobile app showed a rise followed by a decline, peaking in 2020 and gradually decreasing thereafter. With the outbreak of COVID-19 at the end of 2019, offline teaching was restricted, creating a strong demand for online learning across multiple disciplines such as epidemiology, statistics, sociology, and psychology [38]. To address the urgent need for pandemic-related training, a large number of COVID-19 courses were launched on the Guoyuan mobile app, while regular courses were temporarily reduced. Hospitals across Henan Province used the platform for centralized epidemic prevention and control training, resulting in a peak in attendance in 2020. After 2020, as course offerings and clinical activities gradually normalized, attendance levels also returned to the prepandemic baseline. Studies indicate that offline courses outperform online ones in interaction, discussion, and teaching quality [39], while integrating online CME with group learning can significantly enhance doctors’ learning outcomes [40]. The Guoyuan mobile platform mainly offers prerecorded, self-directed courses, where engagement depends on doctors’ initiative. Expanding course coverage and incorporating real-time interaction could improve participation and learning outcomes. Aligned with China’s digital health strategy and the World Health Organization’s framework for lifelong learning, this model illustrates how mobile CME platforms can enhance equitable access and standardize training quality. Although based on a single regional platform, the findings suggest broader applicability. The Guoyuan model provides a scalable approach for strengthening professional competency and continuous education in other Chinese provinces and low- and middle-income countries with limited medical education resources.

### Limitations

Although our findings provide a deep insight into the development of online CME in China, the study still has several limitations. First, the courses released by the Guoyuan education app mainly serve to improve the level of medical personnel in the first stage of promotion, while there are few courses focused on the popularization of medical knowledge among the general population. Future development of the app will enrich the courses’ content and provide more courses on the popularization of basic medical knowledge. Second, considering the assessment requirements of continuing education for medical staff every year in China, we will introduce continuing education courses into the app to enhance the convenience of medical staff to participate in continuing education and expand the influence of the app. Additionally, this study is exploratory in nature, and its findings should be interpreted with certain limitations. The use of a voluntary response sample from a single platform may introduce self-selection bias and limit the generalizability of the conclusions. The analytical outcomes should be interpreted as a preliminary exploration of online CME dynamics. Future studies will use more rigorous sampling frameworks to further validate these exploratory findings and enhance the robustness of the results.

### Conclusions

In this study, we examined the trends in the uptake of mobile online CME services by medical professionals in China through a representative study on a regional platform operated by the National Telemedicine Center of China. The platform started offering medical training via mobile apps in 2018 and is gaining popularity among staff in both tier-3 hospitals and tier-2 and tier‐1 hospitals over the years, particularly after the outbreak of COVID-19. In addition, the platform offered a wide range of courses covering multiple disciplines. Internal medicine was the most popular discipline in terms of the number of available courses, total attendance, and average attendance per course, and ophthalmology and otolaryngology courses had the highest median number of views. The professional title, discipline category, age, and working years were identified as the main factors influencing the satisfaction and recognition of competency enhancement in online CME. To further improve the quality of online CME services, it is necessary to increase the number of courses in disciplines with fewer offerings and conduct research on disciplines with lower satisfaction levels to optimize course structures and provide more targeted content. Additionally, efforts should be made to enhance the user interaction experience of the software platforms, ensuring accessibility and barrier-free usability for individuals across all age groups.

## Supplementary material

10.2196/76299 Multimedia Appendix 1 Major functional modules in the Guoyuan mobile tele-education app.

## Abbreviations

CME: continuing medical education
OR: odds ratio
