# Supplementary material for: The Design and Evaluation of an Online Continuing Medical Education App for Medical Professionals in China: Quantitative Study
Source: JMIR Med Educ. 2026 Feb 23;12:e76299. doi: 10.2196/76299 (PMC12928682; doi:10.2196/76299)
Supplement: Multimedia Appendix 1 [file mededu-v12-e76299-s001.docx]

Major functional modules in the Guoyuan mobile tele-education APP

| Module name | Description |
| --- | --- |
| Lecture recording | The platform will publish recorded lectures for trainees to access. |
| Online lecture | The platform provides online lectures live-streamed to enrolled trainees at the scheduled time. The course instructor could choose to use a third-party video-conferencing platforms (i.e., Zoom and Microsoft Teams) by providing an external link to its students on the platform; or directly uses the live streaming utilities on the platform. |
| Archived publications | Registered users could access and request medical publications archived by the tele-education platform. |
| Courses by discipline | Registered users could browse available courses categorized by disciplines. |
| Discussions | Registered users could discuss on topics related to recent publications, courses, and emerging medical topics. |
| Saved courses | Trainees could save courses to their favorite lists. |
| Recent courses | Users could access the courses they have taken recently. |
| Sharing | Users could share a course, publication, and discussion topics with their friends on other social media platforms (*e.g.,* WeChat). |
| Exams | Scheduled exams for each course could be accessed in this module. |
| Friend circle | A social hub to chat with other users in this platform. |
| Course management | A registered course instructor could manage her/his courses (*e.g.,* schedule lecture, publish exams, open discussion forums) in this module. |
